# Identification of Soluble Mediators in IgG-Mediated Anaphylaxis via Fcγ Receptor: A Meta-Analysis

**DOI:** 10.3389/fimmu.2019.00190

**Published:** 2019-02-12

**Authors:** Audrey Siew Foong Kow, Azirah Chik, Kuan-Meng Soo, Leng Wei Khoo, Faridah Abas, Chau Ling Tham

**Affiliations:** ^1^Department of Biomedical Science, Faculty of Medicine and Health Sciences, Universiti Putra Malaysia, Serdang, Malaysia; ^2^Department of Microbiology and Parasitology, Faculty of Medicine and Health Sciences, Universiti Putra Malaysia, Serdang, Malaysia; ^3^Department of Food Science, Faculty of Food Science and Technology, Universiti Putra Malaysia, Serdang, Malaysia; ^4^Laboratory of Natural Products, Institute of Bioscience, Universiti Putra Malaysia, Serdang, Malaysia

**Keywords:** IgG, anaphylaxis, soluble mediators, meta-analysis, histamine, platelet activating factor, interleukins

## Abstract

**Background:** Anaphylaxis is an acute and life-threatening allergic response. Classically and most commonly, it can be mediated by the crosslinking of allergens to immunoglobulin E (IgE)- high affinity IgE receptor (FcεRI) complex found mostly on mast cells. However, there is another pathway of anaphylaxis that is less well-studied. This pathway known as the alternative pathway is mediated by IgG and its Fc gamma receptor (Fcγ). Though it was not documented in human anaphylaxis, a few studies have found that IgG-mediated anaphylaxis can happen as demonstrated in rodent models of anaphylaxis. In these studies, a variety of soluble mediators were being evaluated and they differ from each study which causes confusion in the suitability, and reliability of choice of soluble mediators to be analyzed for diagnosis or therapeutic purposes. Hence, the objective of this meta-analysis is to identify the potential soluble mediators that are involved in an IgG-mediated anaphylaxis reaction.

**Methods:** Studies related to IgG-mediated anaphylaxis were sourced from five search engines namely PubMed, Scopus, Ovid, Cochrane Library, and Center for Agricultural Bioscience International (CABI) regardless of publication year. Relevant studies were then reviewed based on specific inclusion factors. The means and standard deviations of each soluble mediator studied were then extracted using ImageJ or Get Data Graph Digitiser software and the data were subjected to meta-analysis.

**Results:** From our findings, we found that histamine, serotonin, platelet activating factor (PAF), β-hexosaminidase, leukotriene C4 (LTC_4_), mucosal mast cell protease-1 (MMCP-1), interleukins (IL)-4,−6, and−13; tumor necrosis factor alpha (TNF-α), and macrophage inflammatory protein-1α (MIP-1α) were often being analyzed. Out of these soluble mediators, histamine, PAF, β-hexosaminidase, IL-6, and−13, MIP-1α and TNF-α were more significant with positive effect size and *p* < 0.001. As study effect was relatively small, we performed publication bias and found that there was publication bias and this could be due to the small sample size studied.

**Conclusion:** As such, we proposed that through meta-analysis, the potential soluble mediators involved in rodent IgG-mediated anaphylaxis to be histamine, PAF, β-hexosaminidase, IL-6 and−13 and MIP-1α, and TNF-α but will require further studies with larger sample size.

## Introduction

A rapid and immediate allergic response, anaphylaxis is life-threatening and it affects people from all walks of life (1). Anaphylaxis can be divided into two categories–immunologic and non-immunologic. Immunologically, anaphylaxis can be mediated by the classical IgE-dependent pathway or the alternative pathway also known as the non-IgE-dependent pathway (2).

Anaphylaxis takes place when there is a crosslinking between Igs and their respective receptors found on immune cells. In the classical IgE-dependent pathway, IgE crosslinks with FcεRI that are found on mast cells and basophils causing the degranulation and activation of the cells, and thus releasing soluble mediators that bring about the symptoms of anaphylaxis (3). In the non-IgE-dependent pathway, anaphylaxis can mainly occur due to the involvement of IgG and activation of the complement system (2). In the alternative IgG-mediated pathway, IgG will crosslink with FcγR that are found on a number of immune cells. There are six different types of FcγR, namely FcγRI, RIIA, RIIB, RIIC, RIIIA, and RIIIB whereby all of them have the ability to induce cell activation except FcγRIIB which induces an inhibitory signal (4). In a study by Beutier et al. (5), the FcγRIII receptor was proposed to be the dominant receptor contributing to IgG-dependent passive systemic anaphylaxis induced by IgG_1_, IgG_2a_, and IgG_2b_ antibodies. Macrophages, mast cells, basophils and neutrophils are the immune cells that are involved in the IgG-mediated pathway due to the presence of FcγR on the cells (6). Recently, Beutier et al. (7) have found that platelets are also involved in this pathway, specifically in human FcγRIIA (hFcγRIIA)-induced anaphylaxis. In their newly developed mouse model that expressed only hFcγRIIA, anaphylaxis was expressed through direct activation of aggregrated human IgG on hFcγRIIA-expressing platelets and serotonin is released as a result (7). The non-IgE-dependent pathway can also be activated by complement activation. This happens due to the presence of IgG immunocomplex which can trigger the release of complements C3a, C5a, and C5b-9. These complements will then activate mast cells, basophils and other cells through their specific receptors causing degranulation of the cells and the release of anaphylactic soluble mediators (8).

The immune system is not involved in the activation of non-immunologic anaphylaxis. It happens as a result of direct activation of mast cells as shown by some drug- induced anaphylaxis reactions. This occurs as a result of the activation of human mast cells via a novel G protein coupled receptor (GPCR) identified as MAS-related G protein coupled receptor-X2 (MRGPRX2) (9). The direct activation of the mast cells causes the release of soluble mediators leading to non-allergic anaphylactic reaction (8). Non-immunologic anaphylaxis can also happen as a result of the production of bradykinin as seen in contact system activation. This occurs due to direct or indirect activation of the blood coagulation pathway in IgE-mediated allergic reaction which causes an increase in heparin levels and the activation of factor XII-driven contact system (4).

**Figure 1:**
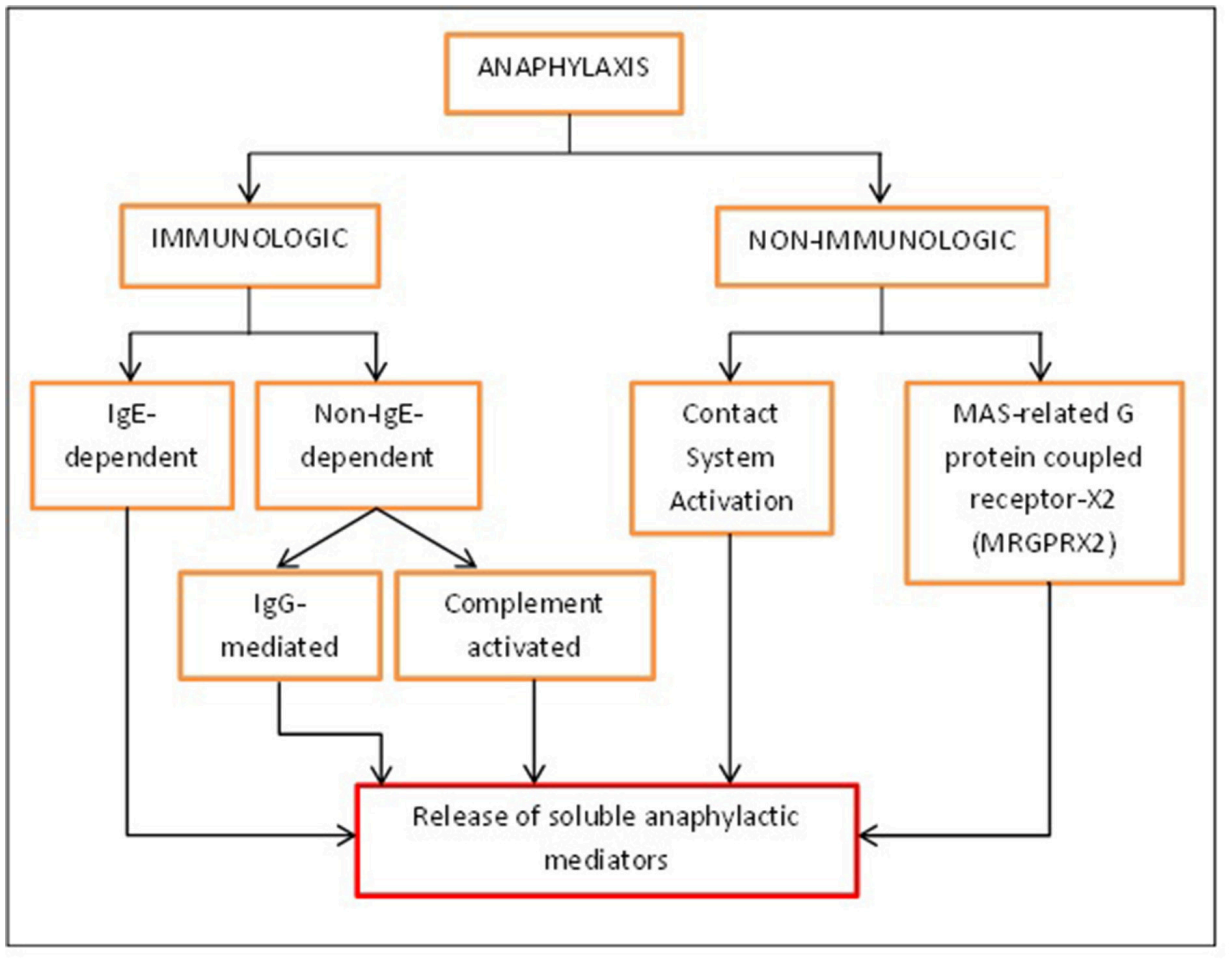
Different activation pathways of anaphylaxis. Anaphylaxis can be activated immunologically and non-immunologically. Immunologically, anaphylaxis can be either IgE-dependent or non-IgE-dependent while non-immunologically; it can be caused by contact system activation or MRGPRX2 found on mast cells. All of these will eventually lead to the release of soluble mediators that will bring about the symptoms of anaphylaxis.

Most reported cases of human anaphylaxis were of IgE nature. However, recently, the alternative IgG pathway was brought into the limelight as there were many cases of reported anaphylaxis in hospitals which were triggered by monoclonal antibodies (mAb) and greatly influenced by IgG instead of IgE. Besides mAb, human IgG-mediated anaphylaxis can also be induced by large quantities of infused dextran, aprotinin, and von Willebrand's factor (5). The IgE-mediated pathway is well-studied compared to IgG-mediated pathway which is still in controversy. IgG-mediated anaphylaxis is postulated to happen in humans as well because of the existence of FcγR on macrophages and their ability to release PAF (10). Even though IgG-mediated anaphylaxis has been proven in rodents, the soluble mediators involved in this pathway vary in different reported findings unlike in IgE-mediated pathway. In the IgE-mediated pathway, key soluble mediators that were usually being studied were histamine and interleukin-4 (IL-4). Histamine, a pre-formed mediator released by mast cells is regarded as the main soluble mediator that induces the IgE-mediated anaphylaxis pathway. Newly synthesized growth factor such as IL-4 is important in the isotype switching of B cells to IgE; hence it's another key soluble mediator being identified in an IgE-mediated anaphylactic reaction (11). The specificity and conclusiveness of the soluble mediators that are involved in IgG-mediated pathway could not be determined as different studies used different type of inducers to identify the responses and effect and it involves different types of cells. Hypothetically, Finkelman has reported the effective soluble mediator involved in this anaphylaxis pathway to be PAF. This soluble mediator is thought to contribute to tachycardia during an anaphylactic attack (12). Due to the variety and inconclusiveness of reported soluble mediators; meta-analysis was done to identify the potential soluble mediators involved in IgG-mediated anaphylaxis for the use of future studies and hopefully with confirmation from larger number of study they could be applied for diagnostic and therapeutic purposes in human IgG-mediated anaphylaxis. (Figure 1)

## Methods

### Search Strategy

Information was obtained from relevant articles that were identified using the keyword of “IgG- or Fcγ-mediated anaphylaxis.” The articles were searched from five databases: PubMed, Scopus, Ovid, Cochrane Library, and Center for Agricultural Bioscience International (CABI). The period of studies was from all years till 2018 and encompassed original papers.

### Eligibility Criteria

**Figure 2:**
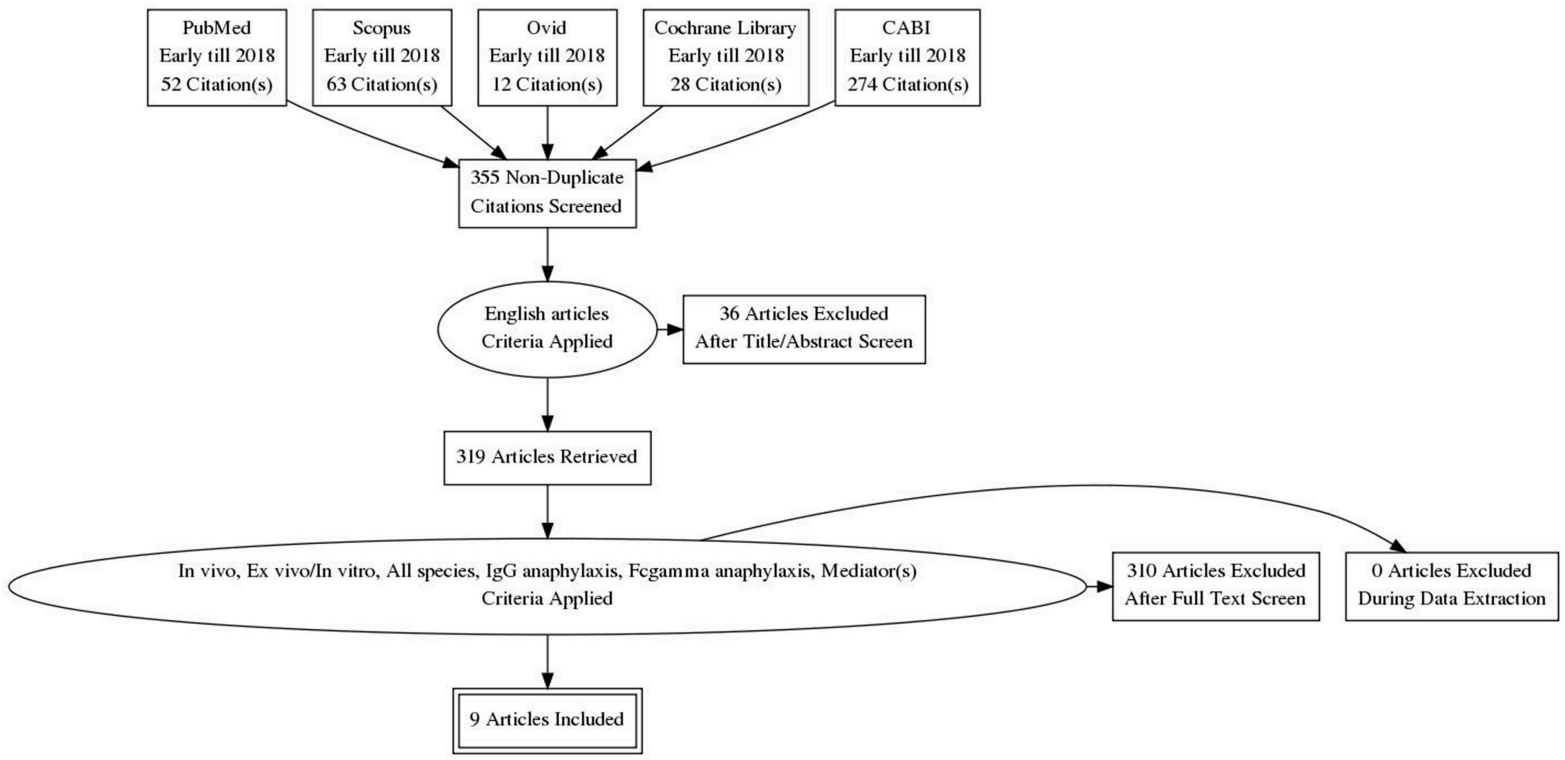
PRISMA flow chart. Only 9 relevant articles were subjected to meta-analysis out of a total of 429 papers retrieved from the five databases. Articles were screened for their relevancy based on the inclusion factors—English article, *in vivo, ex vivo/in vitro*, all species, IgG anaphylaxis, Fcgamma anaphylaxis, mediator(s).

In the current meta-analysis, studies were only included if the related articles consisted of *in vivo* or *ex vivo/in vitro* focusing on induced IgG anaphylaxis pathway regardless of the species. The list of eligibility criteria was shown in Figure 2. All relevant articles containing these criteria were selected regardless of publication year. However, only papers that were published in English were included in this study. Only papers that analyzed the soluble mediator(s) that are released upon anaphylactic induction were included. Besides studies that were specific to IgG-mediated anaphylaxis, studies which comprised both IgE and IgG-mediated anaphylaxis were also included but only data findings related to IgG-mediated anaphylaxis were assessed.

### Study Appraisal and Selection

The screening of relevant articles from literature search was done by assessing the title of article, abstract, the year of publication and also PubMed identification (PMID). Relevant articles were organized according to their title and PMID. Duplicates of study were detected by the same PMID or the same research title, author, and year of publication. These duplicates were excluded and the remaining selected articles were selected using eligibility criteria as mentioned in Figure 2. This was conducted by two reviewers as a confirmation method of study. The full text of selected articles was reviewed for relevant data identification of IgG-mediated anaphylaxis.

### Data Extraction and Organization of Studies

The relevant articles were organized using Excel Worksheet Window version 2007. The papers were screened for relevant data from the literature, graphs, and histograms. The reviewers extracted the data of the number of samples, mean, and standard deviation from the papers that presented the aggregate data, whereas for papers that did not present the aggregate data, the reviewers extracted the data of the levels of mediators of individual samples using methods as follows: (1) Data from graphs were extracted using scatter plot of Get Data Graph Digitiser programme version 2.26.0.20 and (2) data from histogram were obtained using ImageJ software version 150, by measuring the length of bar histogram. The reviewers then calculated the mean and standard deviation using the data of individual samples. All data that involved soluble mediator were extracted.

### Data Analysis

Based on Preferred Reporting Items for Systematic Reviews and Meta-analysis (PRISMA), the data were analyzed for raw mean difference of common soluble mediators involved between non-anaphylactic and anaphylactic groups, according to the method described in a previous study (13). Cumulative mean difference of different soluble mediators was then calculated. The heterogeneity between studies was assessed by using the I-squared index (*I*^2^). Random effect and fixed effect models were used to calculate the cumulative mean difference of studies with significant heterogeneity (*I*^2^ > 75%) and without significant heterogeneity (*I*^2^ < 75%), respectively. Data that were extracted was analyzed using OpenMeta [Analyst] software (Brown School of Public Health, Providence, RI, USA), and the Meta-Essentials program (Erasmus Research Institute of Management, Netherlands). Forest plots were constructed for each soluble mediator and the mean difference value, standard error and *p-values* were obtained. Publication bias was assessed by means of a funnel plot, Egger's regression test, Begg's test, Rosenthal's Fail-Safe test, and unbiased estimate (trim and fill) test.

### Study Outcome

Statistical of weighted mean difference were measured for relevant outcome. The cumulative statistical effects of meta-analysis were analyzed to evaluate studies with high potential to be discussed. Publication bias due to small number of studies was assessed based on the correlation between mean difference and their standard error.

## Results

### Literature Search

The study retrieval and selection were shown in Figure 2. A thorough search inclusive of published articles, book or book chapter, conference paper, and also unpublished articles was done. However, only original papers were selected for meta-analysis. The keywords used as search item were “IgG-mediated anaphylaxis” or “Fcgamma-mediated anaphylaxis.” Search of selected paper was limited to study published in English only from early years to 2018. A total of 429 papers were retrieved from the five databases whereby 52 were from Pubmed, 63 from Scopus, 12 from Ovid, 28 from Cochrane Library, and 274 from CABI. After the removal of duplicates based on the PMID and manual screening of similar titles, 355 papers remained and they were further screened for relevancy based on these inclusion criteria–*in vivo, ex vivo/in vitro*, includes all species and studied the mediators released in IgG-mediated anaphylactic pathway. Non-English papers and papers other than original research articles were excluded in this review. Based on the inclusion and exclusion criteria, only 9 papers were found relevant for this review and used for further analysis. These 9 papers were subjected to meta-analysis based on the criteria that more than one study is needed for each mediator and the data were from two different groups (non-anaphylactic and anaphylactic).

Table 1 depicts the characteristics–type of study and their publication year of the 9 relevant studies being included for meta-analysis. Four (44.4%) of the studies focused solely on the animal model while 77.8% of the studies analyzed the soluble mediators released from different cells. Four (4) studies were published before year 2000 whilst 5 were published after year 2000. This indicated the emerging importance of IgG-mediated anaphylaxis studies in the recent decade.

**Table 1:** Characteristics of relevant studies as categorized based on the source of study, institution and year of publication.

**Category**	**Number of studies**	**Percentage (%)**
**TYPE OF STUDY**		
*In vivo*	4	44.4
*Ex vivo/in vitro*	7	77.8
Both	2	22.2
**YEAR OF PUBLICATION**		
1980 to 1994	1	11.1
1994 to 1999	3	33.3
2000 to 2009	3	33.3
2010 to 2018	2	22.2

### Data Extraction, Organization and Meta-Analysis

**Figure 3:**
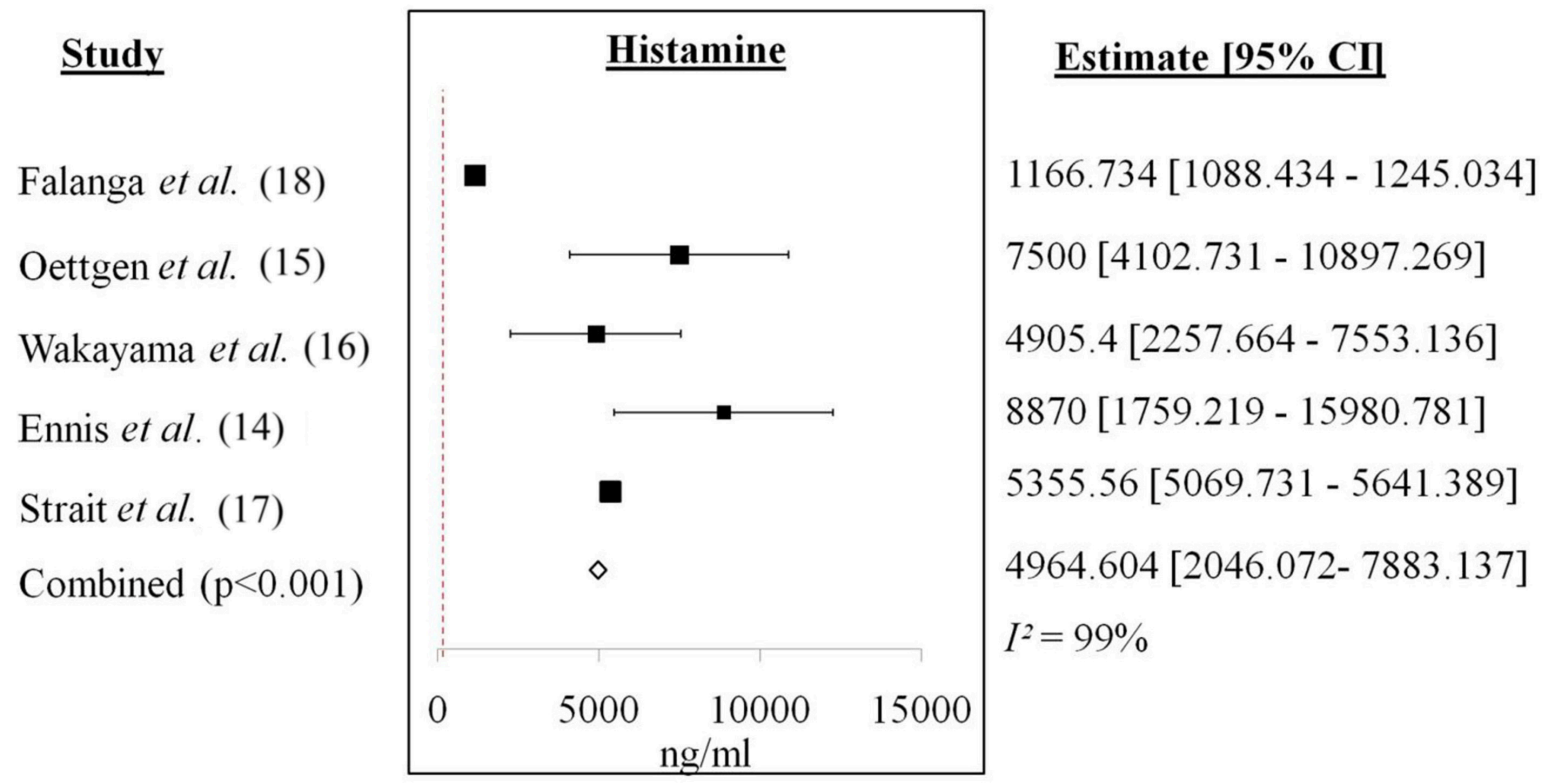
Forest plot of histamine from *in vivo* studies. Increase of histamine level was recorded for each study with a cumulative mean difference of 4964.604 ng/ml.

**Figure 4:**
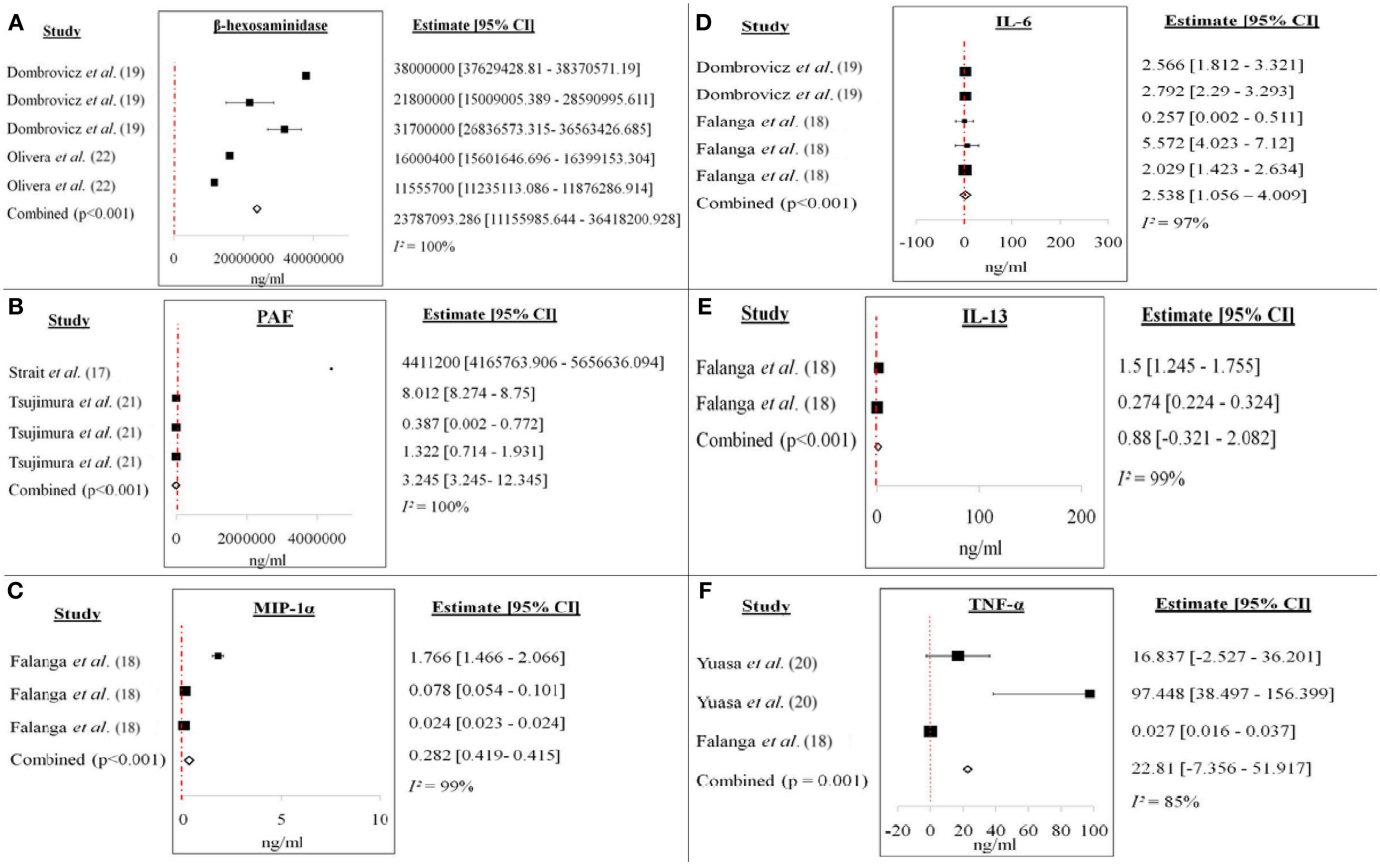
Forest plots of **(A)** β-hexosaminidase, **(B)** PAF, **(C)** MIP-1α, **(D)** IL-6, **(E)** IL-13, and **(F)** TNF-α from *ex vivo/in vitro* studies. Increase of each mediator level was recorded for each study. The cumulative mean difference for each mediator was **(A)** 23787093.286 ng/ml (β-hexosaminidase), **(B)** 3.245 ng/ml (PAF), **(C)** 0.282 ng/ml (MIP-1α), **(D)** 2.538 ng/ml (IL-6), **(E)** 0.88 ng/ml (IL-13), and **(F)** 22.81 ng/ml (TNF-α).

**Figure 5:**
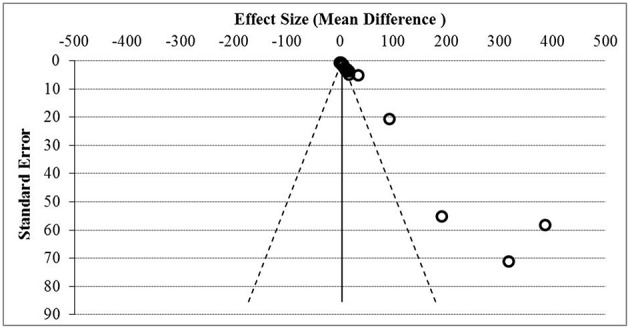
Funnel plot assessment of publication bias. Asymmetrical funnel plot was present indicative of publication bias based on the effect size and standard error.

Data were extracted and organized based on the type of soluble mediator(s) studied, inducer used and the study type as shown in Tables 2, 3. The meta-analysis forest plots for each soluble mediator were as shown in Figures 3, 4, and summarized in Table 4. Publication bias assessment was also evaluated and this was as shown in Figure 5 and Table 5.

**Table 2:** List of *in vivo* studies categorised based on the type of mediator(s) studied, inducer used.

**No**.	**Authors**	**Animal**			**Experiment**	**Inducer**		**Parameter**	**Sample size**	**References**
		**Species**	**Gender**	**Age**		**Sensitize**	**Challenge**			
						**Type of study:** ***In vivo***				
1	Ennis et al., 1983	Guinea pig (Dunkin Hartley)	Male	NS^*^	Active systemic anaphylaxis (triggering IgG antibodies)	OVA (50 mg, subcutaneously) (and 50 mg, intraperitoneally)	OVA [(50 mg, intraperitoneally) on Day 3]; Isolated ventilated lungs was administered with 500 μg OVA in 0.2 mL Krebs' solution as a bolus injection via pulmonary artery	Histamine from lung perfusates	8	(14)
2	Oettgen et al., 1994	IgE-deficient mice (homozygous null mutation of Cεgene)	NS^*^	12 week old	Active anaphylaxis	OVA (100 μg), alum (1 mg) and 300 ng pertussis toxin, intra-peritoneal injection	OVA [500 μg, intravenously (after 18–21 days)]	Plasma histamine (Blood obtained 2 min after challenge)	6	(15)
3	Wakayama et al., 1998	CD40^+/+^ of C57BL/6 background (H-2^b^) mice	NS^*^	NS^*^	Passive systemic anaphylaxis	Purified IgG fraction of anti-OVA serum (5 mg/mouse, intraperitoneally)	OVA after 24 h	Plasma histamine (Blood obtained 2 min after challenge)	6	(16)
4	Strait et al., 2002	BALB/c mice	Female	7–12 week old	Antigen-induced anaphylaxis	1.Affinity-purified GaMD antibody (800 μg/200 μL normal saline, intravenously) 2.GaMD antiserum (200 μL, intraperitoneally)	1.IgG purified from normal goat serum; antigen (100 μg/200 μL normal saline, intravenously) 2.Rat IgG2b anti-mouse FcγRII/III, 2.4G2	1.Serum MMCP-1 (Blood obtained 2 h after challenge) 2.Plasma histamine (Blood obtained 2 min after challenge)	10	(17)
5	Falanga et al., 2012	C57BL/6 × 129sv mice C57BL/6 mice	NS^*^	9 week age (minimum)	IgG-induced passive systemic anaphylaxis	500 μg rat anti-mouse CD16/CD32, clone 2.4G2	None	Serum histamine	21	(18)

* *NS, not stated; GaMD, goat anti-mouse IgD antibody; OVA, ovalbumin; MMCP-1, mouse mast cell protease*.

**Table 3:** List of *ex vivo/in vitro* studies categorised based on the type of mediator(s) studied, inducer used.

**No**.	**Authors**	**Cell**	**Number of cells**	**Experiment**	**Inducer**		**Parameter**	**Sample size**	**References**
					**Sensitise**	**Challenge**			
					**Type of Study:** ***ex vivo/in vitro***				
1	Dombrowicz et al., 1998	BMMCs from 1.BALB/c FcγRIIIα*βγ*_2_ mice 2.BALB/c FcγRIIIαγ_2_ mice	NS^*^	FcγRIII-induced degranulation	2.4G2 antibody (1, 10, 100, 1,000 ng/ml)	Anti-rat IgG (10 μg/ml)	1.β-hexosaminidase	NS^*^	(19)
					2.4G2 antibody (1, 10, 100 ng/ml/10^6^ cells)	None	2.IL-6 (6 h stimulation)		
2	Yuasa et al., 2001	1.Bone marrow-derived mast cells (BMMCs)	5 × 10^5^/m	Mast cell degranulation and mediator release	2.4G (5 μg/ml, 30 min)	F(ab')_2_ fragment of goat anti-rat IgG (0.3–10 μg/ml)	Serotonin (1 h stimulation)	2	(20)
		2.Bone marrow derived mast cells adhered to Swiss 3T3 fibroblast (3T3-BMMCs)	10^5^/sample				1.TNF-α (3 h stimulation) 2.IL-4 (12 h stimulation)	9	
3	Strait et al., 2002	1.Spleen from BALB/c mice	NS^*^	Antigen-induced anaphylaxis	1. Affinity-purified GaMD antibody (800 μg/ 200 μL normal saline, intravenously) 2.GaMD antiserum (200 μL, intraperitoneally)	1.IgG purified from normal goat serum; antigen (100 μg/ 200 μL normal saline, intravenously) 2.Rat IgG2b anti-mouse FcγRII/III, 2.4G2	Spleen PAF (Spleen removed 15 min after challenge)	10	(17)
4	Tsujimura et al., 2008	1.Basophil-containing CD49b^+^ fraction of spleen cells from C57BL/6 mice 2.Basophil-deficient CD49b^−^ fraction of spleen cells from C57BL/6 mice 3.Peritoneal cells from C57BL/6 mice	2 × 10^6^ cells/0.5 ml	IgG1-mediated systemic anaphylaxis	Anti-PenV IgG1 (0.2 mg/ml)	PenV-BSA, (0.4 mg/ml, 37°C, 20 min)	PAF	12	(21)
5	Falanga et al., 2012	1.Mouse bone marrow-derived mast cells	NS^*^	IgG-induced systemic anaphylaxis	Rat anti-mouse FcγRII/III 2.4G2 (10 μg/ml)	Goat anti-rat IgG (10 μg/ml)	1.Histamine and LTC_4_ 1 h stimulation 2.IL-6, IL-13, and MIP-1α (18 h stimulation)	18	(18)
		2.Mouse bone marrow-derived macrophages					1.IL-6 and MIP-1α (18 h stimulation)		
		3.Mouse bone marrow-derived basophil					2.IL-6, IL-13, MIP-1α, and TNF-α (18 h stimulation)		
6	Olivera et al., 2013	Peritoneal mast cells from *S1pr2^−/−^*mice	10^6^ cells	IgG-mediated mast cell degranulation	Rat 2.4G2 anti-mouse CD16/CD32 antibody (10 μg/ml, incubated 1 h, 37 cC)	Goat anti-rat IgG antibody (5 and 10 μg/ml, 30 min, 37°C)	β-hexosaminidase	NS^*^	(22)

* *NS, not stated; PAF, platelet activating factor; IL, interleukin; TNF-α, tumor necrosis factor alpha; MIP-1α, macrophage inflammatory protein-1 alpha; LTC_4_, leukotriene C4*.

**Table 4:** Compilation of the cumulative mean difference, *p-value* and 95% confidence level (CI) of each mediator studied in both *in vivo* and *ex vivo/in vitro* studies of IgG-mediated anaphylaxis between non-anaphylactic and anaphylactic groups. *p*<*0.05* is considered significant.

**Mediators**	**Number of studies**	**Heterogeneity**	**Effect size**		**95 % CI**	
			**Cumulative mean difference (ng/ml)**	***p*-value**	**Upper value**	**Lower value**
***IN VIVO*** **STUDY**						
Histamine	4	99%	4964.6	<0.001	7883.137	2046.072
***EX VIVO/IN VITRO*** **STUDY**						
β-hexosaminidase	2	100%	23787093.286	<0.001	36418200.928	11155985.644
PAF	2	100%	3.245	<0.001	12.345	3.245
MIP-1α	1	99%	0.282	<0.001	0.419	0.415
IL−6	2	97%	2.538	<0.001	4.009	1.056
IL−13	1	99%	0.88	<0.001	2.082	−0.321
TNF-α	2	85%	22.81	0.001	51.917	−7.356

**Table 5:** Summary and conclusion of publication bias assessment from asymmetry of funnel plot, Egger's regression test, Begg's test, Rosenthal's Fail-safe N, and Trim and Fill (unbiased estimate) analysis.

**Type of study**	**Asymmetry of funnel plot**	**Egger's regression test**	**Begg's test**	**Rosenthal's fail-safe N**	**Trim and fill (Unbiased estimate)**	**Conclusion**
Mediator(s)	Present	*p < 0.05*	*p < 0.05*	*N* = 2659	5.59 (10.16–85.33)	Publication bias: Yes

Tables 2, 3 showed specific characteristics of study that were selected to proceed with meta-analysis. All potential soluble mediators studied and the inducers used to induce IgG-mediated pathway of anaphylaxis were listed. All studies were divided into *in vivo* (Table 2) and *ex vivo*/*in vitro* (Table 3).

Figure 3 showed the forest plot of histamine from *in vivo* studies. All the studies showed increment in the histamine level released between anaphylactic groups compared to non-anaphylactic groups after being induced by IgG inducers. Results were of mean weighted difference between the two groups.

Figure 4 showed the forest plots of (A) β-hexosaminidase, (B) PAF, (C) MIP-1α, (D) IL-6, (E) IL-13, and (F) TNF-α of *ex vivo/in vitro* studies. All the studies showed increment in the soluble mediators released in the anaphylactic groups compared to non-anaphylactic groups after being induced by IgG inducers. Results were of mean weighted difference between the two groups.

Table 4 summarized the cumulative mean difference, *p-value* and 95% confidence level of each soluble mediator studied in all study types of IgG-mediated anaphylaxis. The cumulative mean differences between non-anaphylactic and anaphylactic groups showed increase in the levels of soluble mediators released after being challenged by inducers of IgG-mediated anaphylaxis. Based on the *p-values*, significant soluble mediators in IgG-mediated anaphylaxis were histamine (*in vivo*) [4964.6 ng/ml; *p* < *0.001*] and in *ex vivo/in vitro*–β-hexosaminidase (23787093.286 ng/ml; *p* < *0.001*), PAF (3.245 ng/ml; *p* < *0.001*), MIP-1α (0.282 ng/ml; *p* < *0.001*), IL-6 (2.538 ng/ml; *p* < *0.001*), and IL-13 (0.88 ng/ml; *p* < *0.001*), and TNF-α (22.81 ng/ml; *p* = *0.001*). *p* < *0.05* is considered significant. Although MMCP-1, serotonin, IL-4, and LTC_4_ were also being studied, meta-analyses could not be performed for these mediators due to insufficient data as there was only one study, respectively, that analyzed these soluble mediators.

Figure 5 showed the funnel plot assessment of publication bias based on the effect size and standard error, and Table 5 summarized the general agreement of results from five methods of publication bias assessment. Publication bias assessment was analyzed due to the small study effect. This was to rule out that the positive results of all the soluble mediators studied were not only from studies that reported significant results but encompasses all results. From the assessment, it was found that publication bias due to small study effect was present with Egger's regression test and Begg's test *p* < 0.05.

## Discussion

The identification of potential soluble mediators involved in IgG-mediated anaphylaxis was important for diagnostic and therapeutic purposes as the involvement of IgG has been reported in several cases of clinical anaphylaxis whereby serum tryptase and IgE were not detected (6). Key soluble mediators such as histamine and IL-4 were commonly being studied in IgE-mediated anaphylaxis but there is no consensus on the soluble mediators for IgG-mediated anaphylaxis. This is possibly because IgG-mediated anaphylaxis can be activated by different cells. Due to the variation of cell types, in this present study we analyzed all potential soluble mediators that were involved using meta-analysis. Meta-analysis is a statistical method employed to study the effectiveness of a treatment intervention by combining the data from two or more studies (23). It provides a numerical value that indicates whether the treatment was beneficial or otherwise as explained by Israel and Richter (23). In our meta-analysis, our approach was not on the effectiveness of a treatment but rather on the soluble mediators that are released upon induction of IgG-mediated anaphylaxis. It analyzed the potential soluble mediators that were being studied and showed significant difference between the non-anaphylactic and anaphylactic group.

Based on the literature search, IgG pathway of anaphylaxis could be activated by a number of inducers as stated in Tables 2, 3. The inducers used include 2.4G2 antibody, ovalbumin, and specific IgG antibodies. The levels of most reported soluble mediators in anaphylactic groups showed significant increase compared to non-anaphylactic groups regardless of the type of inducers used. Soluble mediators of anaphylaxis are subdivided into three categories known as (a) pre-formed mediators released by degranulation, (b) newly synthesized pro-inflammatory lipid mediators, and (c) newly synthesized growth factors, cytokines, and chemokines (24). The most frequent soluble mediators being studied in IgG-mediated anaphylaxis were histamine and MMCP-1 *in vivo* (Table 2); histamine, PAF, β-hexosaminidase, serotonin, LTC_4_, IL-4,−6 and−13, TNF-α, and MIP-1α *ex vivo/in vitro* (Table 3). Only selected soluble mediators were subjected to further meta-analysis as meta-analysis requires the comparison of more than one study to be valid and include data from two groups (in our study non-anaphylactic group and anaphylactic group).

We identified histamine, β-hexosaminidase, PAF, MIP-1α, IL-6 and−13, and TNF-α as potential soluble mediators that are produced by IgG-mediated anaphylactic animals or cells based on the significant *p-values* (<0.05) of each soluble mediator between the non-anaphylactic and anaphylactic groups (Table 4). The cumulative mean difference indicates that the levels of these soluble mediators in the anaphylactic group were higher than in the non-anaphylactic group as shown in the forest plots of each soluble mediator analyzed (Figure 3). The increase of these pro-inflammatory soluble mediators serves as an indication of an anaphylactic reaction. Histamine is mostly being analyzed in an *in vivo* system while β-hexosaminidase, PAF, MIP-1α, IL-6 and−13, and TNF-α were being analyzed in *ex vivo*/*in vitro* system from different cells.

Histamine, with a cumulative mean difference of 4964.6 ng/ml is the main soluble mediator of IgE-mediated anaphylaxis and it also contributes to IgG-mediated anaphylaxis. Histamine is a pre-formed soluble mediator that is stored mainly in mast cells. Mast cells has long been regarded as the main effector cell involved in IgE-mediated anaphylaxis, however, Oettgen et al. (15) has demonstrated that anaphylaxis could also be activated in IgE-deficient mice by non-IgE dependent mechanisms. This is because murine mast cells also express FcγRIIb1, FcγRIIb2, and FcγRIII IgG receptors and the degranulation of mast cell and release of histamine is brought about by the crosslinking of IgG to the FcγRIII (25). Histamine causes coronary vasoconstriction, cardiac depression, systemic vasodilatation and tachycardia, inhibits release of norepinephrine, chemotaxis and release of other soluble mediators by inflammatory cells during anaphylaxis (11). Another pre-formed inflammatory soluble mediator of interest was β-hexosaminidase (cumulative mean difference: 23787093.286 ng/ml) which is also found in the granules of mast cells (26). β-hexosaminidase is released at the same time as histamine and it is a typical indicator of mast cell degranulation (27). Similar to histamine, β-hexosaminidase is able to induce the synthesis of arachidonic acid metabolites, cytokines, and chemokines which will lead to anaphylactic symptoms (26). Huang et al. (26) also highlighted that β-hexosaminidase as a better soluble mediator in determining mast cell degranulation because of its slower release and better retention in the system compared to histamine which has a short half-life of about 1 min.

A potent phospholipid-derived soluble mediator, PAF (cumulative mean difference: 3.245 ng/ml) is synthesized mainly by macrophages (10) and other cells–basophils, neutrophils (1) and mast cells (12). It is produced within minutes of aggregation and it contributes to the amplification and prolongation of anaphylaxis (28). Levels of PAF are widely being measured likely because of its parallel correlation to the severity of anaphylaxis (presumably IgE-mediated) as documented by Vadas et al. (29). The mean serum PAF levels were much higher in anaphylactic patients than control patients with an elevation from 4 (control) to 71% in anaphylactic patients of grade 2 anaphylaxis (29). Besides being an indicator of severity of anaphylaxis, Finkelman (12) stated that PAF plays a role in the decreased of myorcardial function in IgG-mediated anaphylaxis whereas this was caused by histamine in IgE-mediated anaphylaxis. Apart from that, PAF also contributes to anaphylactic shock (12); decreased coronary blood flow, increased activation and recruitment of neutrophils and eosinophils, induces local and systemic platelet aggregation, peripheral vasodilatation, and severe hypotension (11).

Soluble mediators such as IL-6 and−13 and TNF-α are newly synthesized growth factors (30). These are released by other immune cellular cells upon being triggered by early phase soluble mediators and are involved in the late phase of an anaphylactic reaction enhancing the anaphylactic reaction (26). A pleiotropic mediator (31), IL-6 (cumulative mean difference: 2.538 ng/ml), can be synthesized and secreted by monocytes, T-cells, fibroblasts and endothelial cells (32). IL-6 plays a role in acute phase reactions, chronic inflammation, autoimmunity, endothelial cell dysfunction, and fibrogenesis (31). Even though there may be differences between anaphylaxis in murine and humans, Stone et al. (30) found that the elevated serum IL-6 in human IgE-anaphylaxis correlated with the anaphylactic symptom of hypotension. In a study by Strait et al. (33), they found that IL-13 (cumulative mean difference: 0.88 ng/ml) could worsen anaphylaxis as they are able to increase sensitivity to vasoactive mediators through vascular leak. In their study, mice pre-treated with IL-13 and then challenged with FcγRIII entered a stage of severe shock showing signs of hypothermia, decreased activity and death (33). IL-13 is able to activate the same signal transduction pathways as IL-4 hence induces the production of IgE and also class-switching to IgG_4_ together with CD40 stimulation (33). TNF-α with a cumulative mean difference of 22.81 ng/ml is also an inflammatory soluble mediator and is released as both pre-formed and late-phase mediator and has the ability to activate neutrophils, recruits other effector cells and enhances the synthesis of chemokine (34). Primarily synthesized by macrophages, astroglia, microglia, Langerhans cells, Kupffer cells, and alveolar macrophages, TNF-α is a powerful pro-inflammatory soluble mediator (35). Kang et al. (36) have successfully demonstrated that the involvement of TNF-α in the late phase of anaphylactic reactions was through the activation of cPLA_2_ by activating the p38 MAPK and ERK pathways. Previously this same research group found that TNF-α which was produced by PAF-mediated nuclear factor-κB activation, played a role in the late phase of anaphylactic reactions (37).

Chemokine such as MIP-1α (cumulative mean difference: 0.282 ng/ml) together with mediators has a role in driving the late phase reaction of allergic diseases (38). Upon stimulation, MIP-1α can be secreted by a number of cells such as monocytes, T lymphocytes, B lymphocytes, neutrophils, dendritic cells, and NK cells (39). Although mainly important in the late phase reaction of allergic diseases, Miyazaki et al. (38) discovered that the expression of MIP-1α is needed for optimal mast cell degranulation which is the hallmark of allergy. They reported that the expression of MIP-1α was rapidly induced in their murine model of allergic conjunctivitis (38). This further supported the works of Laffargue et al. (40) whereby MIP-1α were found able to increase the influx of calcium and degranulation of mast cells as shown by the release of β-hexosaminidase.

Due to the small effect size in our meta-analysis, publication bias was assessed to rule out the possibility that the reported increase of soluble mediator levels were not biased. Publication bias is defined as “any influence that reduces the amount of good science appearing in the literature” (41). It can arise due to poor quality in the research design, small sample size, external funding, negative findings, failure of authors to submit manuscripts, or rejection by journal editors as stated by Scholey and Harrison (41). From our assessment it was found that publication bias existed in our review as measured by the standardized mean difference using Hedge's g measurement in relation to standard error of study. Both Begg's and Egger regression tests showed statistical significance with *p* < 0.05 indicating that publication bias was affected by the small effect size. The funnel plot also showed an asymmetric distribution of studies and further test of Rosenthal's fail-safe N showed an overall Z-score above zero which confirmed that combined effect size was statistically significant. Although small sample size may decrease the power of respective study but it should not be left out as it might contain important information for the data extraction as stated by Turner et al. (42). The small number of study in our meta-analysis is due to the specific focus of our study whereby we focused on IgG-mediated anaphylaxis pathway and the soluble mediators that were released upon induction. Although there were many studies on IgG-mediated anaphylaxis, their focus were on the measurement of rectal temperature, the types of Fcγ receptors involved and the types of cells involved. Some papers also did not present data from the non-anaphylactic groups; hence we could not perform meta-analysis. In addition, some of the soluble mediators analyzed were from one study only which could not be assessed by meta-analysis too. Even though the non-IgE-dependent pathway can be activated by the complement system, our focus was more toward the IgG-dependent pathway. Hence, we did not include studies that involved the complement system. Our meta-analysis on the soluble mediators released only took into account of the direct activation by specific IgG antibodies, high dose of OVA or 2.4G2 antibody which targets the FcγRII/RIII receptors. Even though our meta-analysis is from a small number of studies, it is hoped that our findings will be able to draw the attention of more researchers working in this area to fill in the research gap.

### Limitation

The data that we have used in this study contained several limitations which have contributed to publication bias in our meta-analysis. Firstly, the focus of our meta-analysis is very specific whereby only papers that studied the released of mediators were included. In a previous study by Khodoun et al. (6), they found that the non-soluble mediator/blood marker neutrophil could be used to indicate the activation of IgG-mediated anaphylaxis in human. The expression of FcγRIII showed a decrease without an increase in IL-4Rα expression in their human neutrophils cultured with IgG immune complexes. However, it is time consuming and technically challenging to detect the FcγRIII and IL-4Rα expression on cells from patients. As anaphylaxis happens rapidly and is life-threatening, it would be more ideal if any of these biomarkers could be identified in an easier and faster manner. Hence, the identification of potential soluble mediators for IgG-mediated anaphylaxis was specifically the focus of our study as the detection of soluble mediators in blood is relatively rapid and easy. Although there were many papers which studied IgG-mediated anaphylaxis, their data were on rectal temperature changes, analysis on the type of Fcγ receptors involved or the types of cells involved. In addition, only inducers that specifically induced the IgG-anaphylaxis pathway−2.4G2, high dose of OVA and specific IgG antibodies were included in our meta-analysis. Apart from that only studies published in English were included in this meta-analysis and there may be strong studies from non-English publication that had been excluded. Other than that studies that focus on IgG-mediated anaphylaxis is still in its early stage compared to IgE-mediated anaphylaxis which is better studied; it was noted that there was lesser focus on IgG-mediated anaphylaxis until recently as the focus was mainly on IgE-mediated anaphylaxis which was considered the main cause of anaphylaxis.

## Conclusion

In conclusion, our meta-analysis found that the potential mediators involved in IgG-mediated anaphylaxis in rodents to be histamine, β-hexosaminidase, PAF, IL-6 and IL-13; MIP-1α and TNF-α with a reasonable cumulative effect value. These proposed potential mediators could be used in future animal studies of IgG-mediated anaphylaxis and hopefully with confirmation from larger number of study they could be applied for diagnostic and therapeutic purposes in human IgG-mediated anaphylaxis.

## Author Contributions

The analysis was done by AC (first reviewer) and AK (second reviewer) confirmed them. Both AK and AC contributed equally on the preparation of this manuscript. Manuscript was reviewed and proofread by K-MS, FA, and LWK. CLT conceived the idea, reviewed the drafts, and provided important information for the completion of this manuscript.

### Conflict of Interest Statement

The authors declare that the research was conducted in the absence of any commercial or financial relationships that could be construed as a potential conflict of interest.
